# Head shaking does not alter vestibulo ocular reflex gain in vestibular migraine

**DOI:** 10.3389/fneur.2022.967521

**Published:** 2022-09-30

**Authors:** Priyani Patel, Patricia Castro, Nehzat Koohi, Qadeer Arshad, Lucia Gargallo, Sergio Carmona, Diego Kaski

**Affiliations:** ^1^Adult Diagnostic Audiology Department, University College London Hospitals, London, United Kingdom; ^2^The Ear Institute, Faculty of Brain Sciences, University College London, London, United Kingdom; ^3^Department of Brain Sciences, Imperial College London, London, United Kingdom; ^4^Universidad del Desarrollo, Escuela de Fonoaudiologia, Facultad de Medicina Clinica Alemana, Santiago, Chile; ^5^Department of Clinical and Movement Neurosciences, Centre for Vestibular and Behavioural Neuroscience, Institute of Neurology, University College London, London, United Kingdom; ^6^inAmind Laboratory, Department of Neuroscience, Psychology and Behaviour, University of Leicester, Leicester, United Kingdom; ^7^Fundación San Lucas para la Neurociencia, Rosario, Argentina; ^8^Cátedra Neurofisiología de la Universidad Nacional de Rosario, Rosario, Argentina

**Keywords:** vestibular migraine, vestibulo ocular reflex, head shake, motion sensitivity, vHIT

## Abstract

Vestibular Migraine (VM) is the most common cause of non-positional episodic vestibular symptoms. Patients with VM commonly report increased motion sensitivity, suggesting that vestibular responses to head movement may identify changes specific to VM patients. Here we explore whether the vestibulo-ocular reflex (VOR) gain alters in response to a clinical “headshake” maneuver in patients with VM. Thirty patients with VM in the inter-ictal phase, 16 patients with Benign Positional Paroxysmal Vertigo (BPPV) and 15 healthy controls were recruited. Patients responded to the question “Do you feel sick reading in the passenger seat of a car?” and completed a validated motion sickness questionnaire as a measure of motion sensitivity. Lateral canal vHIT testing was performed before and after headshaking; the change in VOR gain was calculated as the primary outcome. Baseline VOR gain was within normal limits across all participants. There was no significant change in VOR gain after headshaking in any group (*p* = 0.264). Patients were 4.3 times more likely to be in the VM group than in the BPPV group if they reported nausea when reading in the passenger seat of a car. We postulate that a headshake stimulus may be insufficient to disrupt cortical interactions and induce a change in VOR gain. Alternatively, changes in VOR gain may only be apparent in the acute phase of VM. Reading in the passenger seat of a car was considered uncomfortable in all VM patients suggesting that this specific question may be useful for the diagnosis of VM.

## Introduction

Vestibular Migraine (VM) is the most common cause of non-positional episodic vestibular symptoms, affecting 1.4% of the general population (1). The migrainous symptoms of VM include recurrent headaches with heightened sensitivity to sensory stimulus, nausea and/or vomiting (2) with recurrent episodic vertigo attacks lasting minutes to several hours (3). The pathophysiology of VM is incompletely understood and the diagnosis is based on clinical features as there are no objective biomarkers to provide a definitive diagnosis (4).

From a clinical perspective, there is a need for an objective marker for VM that can be applied in the clinical setting. A sensitive test for VM would enable clinicians to differentiate between VM and other vestibular disorders with overlapping symptoms (e.g., Meniere's disease). Moreover, a clinical biomarker would facilitate an earlier diagnosis of VM, a major limitation of the current diagnostic criteria that require at least five episodes for diagnosis (3); thus, patients may be underdiagnosed and consequently inappropriately treated when presenting with the first few attacks.

The development of clinical biomarkers may be informed by the nature of patient-reported symptoms in VM. Thus, patients with VM commonly describe excessive motion sensitivity (5, 6), suggesting a role for impaired visuo-vestibular interactions as an underlying mechanism of VM symptoms (7). Other hypotheses to account for vestibular symptoms experienced by VM patients include abnormalities in the integration of inner ear semi-circular canals (SCC) and otolith afferents, and between these and other sensory modalities (e.g., vision) (8), a mechanism that may account for increased head movement induced dizziness and disorientation in VM (9).

A clinical test involving head movement is head-shaking nystagmus (HSN) (10, 11). Here, the head is tilted forward 30 degrees and rotated to the left and right in the yaw plane in order to elicit subclinical vestibular abnormalities by activation of the velocity storage mechamsim that prolongs the time constant of the rotational vestibulo-ocular reflex (VOR) (10–12). This test is abnormal mostly in peripheral disorders, however, abnormalities have been described in central disorders, including “perverted” HSN (13–16) and in patients with recurrent spontaneous vertigo with interictal HSN—where horizntal HSN nystagmus can be found in the absence of peripheral dysfunction (17). Central HSN may arise from an assymetry in the velocity storage mechanism, central gain or the central adaptation, or from an abnormal cross-coupling of velocity storage pathways (18).

It is a recognized finding that patients with VM may have abnormally elevated VOR (nystagmic) responses to water caloric irrigation (19, 20). This is thought to be due to a hyper-excitable vestibular network leading to increased motion sensitivity. To our knowledge changes in vestibular excitability that can be induced by head shaking has not been previously explored. Here we explore the change in VOR gain using the video head impulse test (vHIT) following a clinical “headshake” maneuver in patients with VM. We also explore the frequency of motion sensitivity in VM and BPPV as measured using questionnaires.

## Methods

Patients with VM and healthy controls (HC) were invited to participate. Participants were recruited from neuro-otology clinics in the United Kingdom and Argentina from 1st November 2020 to 30th June 2021. All patients were diagnosed by a Neurologist with expertise in VM (DK and SC) and met the criteria for VM by Bárány (3) or the third edition of the International Classification of Headache Disorders (21). Patients with benign paroxysmal positional vertigo (BPPV) were recruited as a disease control, following first diagnosis, prior to treatment repositioning maneuvers, and in accordance with established diagnostic criteria (22). VM participants were on various prophylaxis medications. All participants had an attack within the last year and were tested in the inter-ictal phase.

Participants with eye movement abnormalities, ocular pathologies affecting detection of pupils, or neck problems that could interfere with the headshake were excluded. In addition, patients with an overlap of two or more diagnoses were not included in this study.

Sample size calculation was performed using the outcomes and effect sizes from Bednarczuk et al. (7). The authors found a significant increase in rotation thresholds in VM patients following prolonged optokinetic stimulation. When using the effect size found in this study, for a power of 80% and a significance of 0.05, the required sample size was 4. Given the small estimated sample size required in view of the expected variability in neurophysiological outcomes in patients with VM, 30 patients with VM were recruited.

Before being tested, we evaluated patients' motion sickness susceptibility by asking: “Do you feel sick reading in the passenger seat of a car?” and asked them to complete the Motion Sickness Susceptibility Questionnaire (MSSQ).

vHIT testing, prior to head shaking (PRE-HS) was performed for the right and left lateral semicircular canal in each ear independently. A minimum of 20 head thrusts with a head displacement of 10–20 degrees, and a range of peak head velocities (150–200 deg/s) and peak head acceleration (1,200–2,500 deg/s) were obtained for each lateral semicircular canal. The VOR gain was defined as the sum of eye movement velocity divided by the sum of head movement velocity during head turn. The normal VOR gain value is 1.0 which means there is compensatory eye velocity which is equal to head velocity in the opposite direction (23).

**Figure 1:**
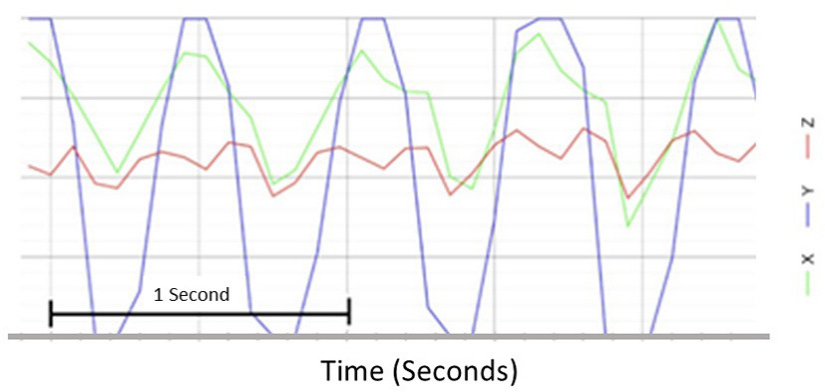
Representative trace of a 2 Hz head shaking using the accelerometer in a graph format. Headshake stimulus using an accelerometer was measured in the y-axis.

Immediately following vHIT testing, a standard clinical headshake was carried out. Participants were asked to close their eyes and tilt their head downward by 30 degrees to ensure their lateral canals were on its horizontal axis; the participant's head was then passively oscillated from side to side at a frequency of 2 Hz for a total of 30 s. To ensure there was no variability in headshake between participants, an accelerometer was used to control and measure each headshake (Figure 1) secured over the participant's occiput using a tight velcro strap that did not interfere with the vHIT setup. Immediately following headshake, a second vHIT (post head shaking, POST-HS) to test lateral SCCs was performed. Participant instructions, test set-up and vHIT testing parameters remained the same as for the PRE-HS vHIT.

### Data analysis

Data was analyzed using Shapiro-Wilk test to identify the distribution. A normal distribution was observed so parametric testing was used. Change in VOR gain was calculated as POST-HS VOR gain subtracted from PRE-HS VOR gain. Bonferroni correction *post hoc* analysis was used. Significance was considered when *p* < 0.05. All statistical analysis was performed using Statistical Package for Social Sciences (SPSS, Version 27, IBM Corporation, NY). Paired sample *t*-test was used to compare pre- and post-VOR gain. Two-way mixed model analysis of variance (ANOVA) was used to compare the means of VOR gain pre vs. post headshake between VM, BPPV group, and HC group. To compare the change in VOR gain between pre- and post- between groups a one-way ANOVA was performed.

## Results

Thirty patients with VM (mean age 48.3, age range 28–67, 22 female/8 male), 16 participants with BPPV (mean age 52.1, age range 31–66, 10 female/6 male), and 15 healthy controls (mean age 43.0, age range 27–65, 10 female/5 male) were recruited for this research study, following informed written consent. All participants in the three groups (VM, BPPV, and HC) had baseline VOR gain within normal limits. Nevertheless, BPPV patients had significantly higher mean VOR gain compared to the VM patients (*p* < 0.01 for PRE-HS, and *p* < 0.001 for POST-HS) that also trended toward significance with the HC (*p* = 0.067 for PRE-HS, and *p* = 0.068 for POST-HS), in keeping with previous reports (7).

**Figure 2:**
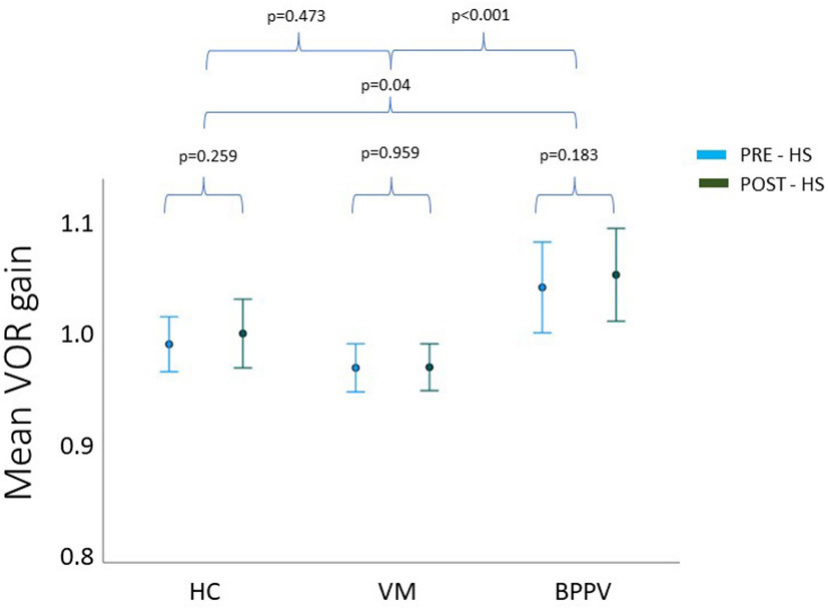
Mean pre and post headshake VOR gain for each group. HC, healthy controls; BPPV, benign paroxysmal positional vertigo; VM, vestibular migraine.

Repeated measures ANOVA showed no statistically significant interaction between time^*^group on VOR gain PRE- and POST-HS (*p* = 0.711). The main effect of time was not statistically different for mean VOR gain at the different time points (PRE- and POST-HS) (*p* = 0.264) (Figure 2). As there was no effect of headshake within groups (VM, BPPV, HC), the PRE- and POST-HS VOR gains were grouped to provide a total mean vHIT VOR gain value per group.

One way ANOVA revealed a statistically significant difference in the total mean VOR gain between the three groups (*F* = 9.725, *p* < 0.001). *Post hoc* analysis showed this was driven by a difference between the BPPV and VM group (*p* < 0.001), and this time the difference between BPPV and HC was also significant (*p* = 0.04). Again, there was no statistical significance between the VM and HC groups (*p* = 0.473).

The change in VOR gain was calculated by subtracting the VOR gain POST-HS from VOR gain PRE-HS. In all three groups, this value showed an increase in mean VOR gain POST-HS compared to PRE-HS. The VM group had the widest range of VOR gain change, while the other two groups showed similar dispersion. One-way ANOVA however found no significant differences between groups in the VOR gain change (*F* = 0.343, *p* = 0.711).

Regarding the subjective measures, all patients with VM referred feeling sick when reading in the passenger seat of a car, while none of the HC and only 9 patients with BPPV replied positively to this question. We calculated the odds ratio of a patient being in the VM group when replying “yes” to this question with a result of patients being 4.3 times more likely to be in the VM group than in the BPPV group. However, patients with VM did not have significantly higher scores in the MSSQ compared to BPPV patients (*p* = 0.92). Additionally, the MSSQ score did not correlate with the change in VOR gain after headshake (r = 0.17, *p* = 0.91).

## Discussion

We show that patients with VM do not have heightened VOR gain immediately following headshake compared to patients with BPPV or healthy controls. Our data confirms previous reports of heightened VOR gain in patients with BPPV who have elevated VOR gain (here both PRE- and POST-HS) compared to VM and healthy controls (7).

Headshaking was used in our paradigm to generate a change in vestibular network excitability which could be non-invasively measured using a simple bedside test. We postulate that a clinical headshake stimulus may be insufficient to disrupt cortical interactions and induce a change in VOR gain. This study delivered a stimulus of 2 Hz for 30 s, following clinical headshaking nystagmus test protocols (10, 11). Although this stimulus seems the most appropriate as it is already clinically applicable, it would be of interest to discern whether changing the frequency, duration, or amplitude of the headshake could induce greater changes to the VOR gain. However, VM patients are sensitive to head movements so performing a faster headshake may not be tolerable for patients, thus limiting its use clinically (24). Alternative stimuli, such as moving visual stimuli may alter vestibular excitability thresholds via modulation of visuo-vestibular interactions, without necessitating head movements. Visual-vestibular interactions in patients with a pre-existing peripheral vestibular disorder has been investigated previously (7), where VOR thresholds were significantly increased following visual motion exposure of 5 min in VM patients compared to migraine patients (without vestibular symptoms) and BPPV patients. These findings support the concept that visual stimulation alters normal visual-vestibular network function in patients with VM and lend further support to the notion that assessing vHIT pre and post prolonged visual motion may be a suitable candidate as a possible biomarker, although of lesser practicality, than the headshake employed in this study.

**Figure 3:**
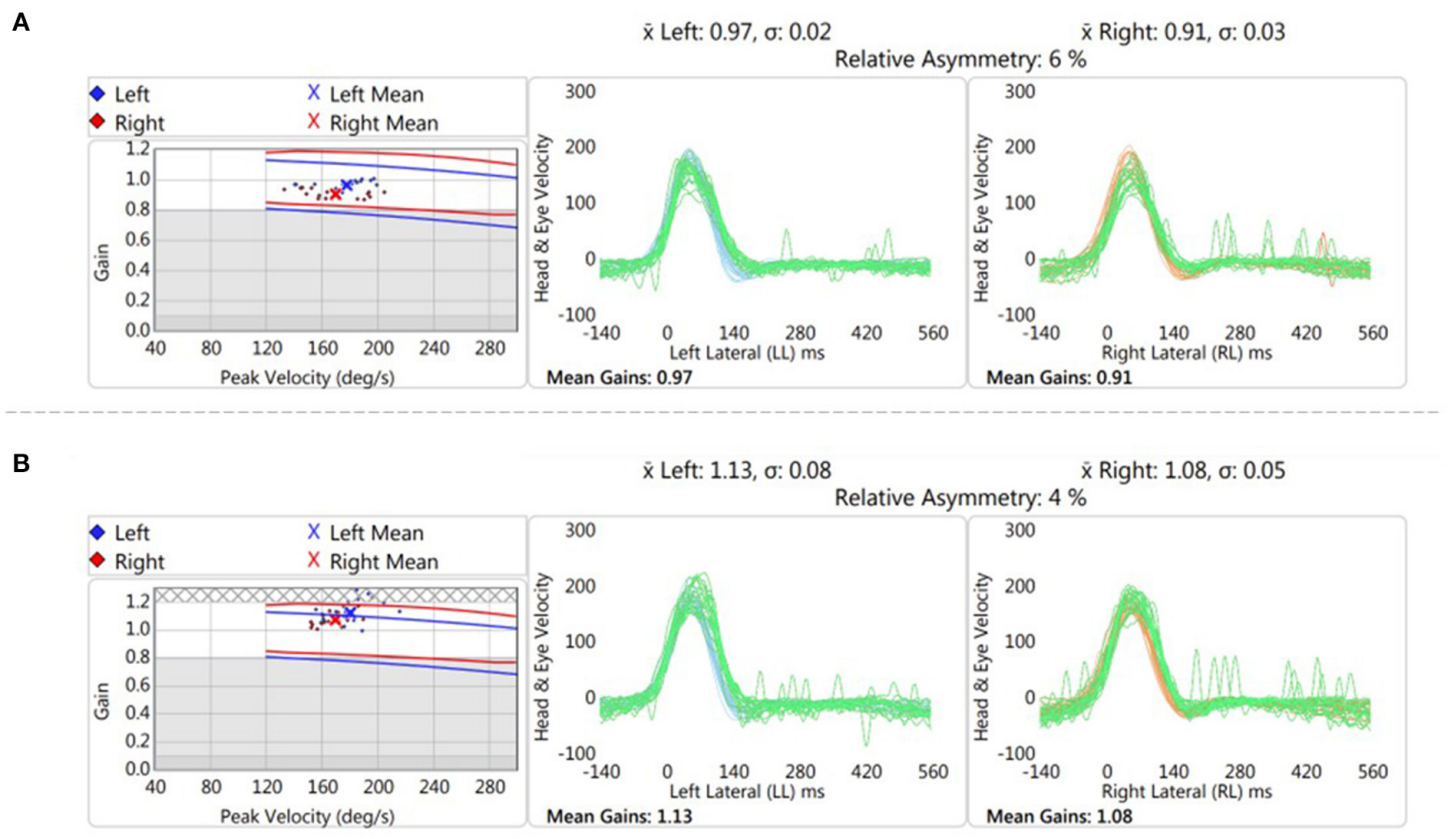
Representative trace of vHIT testing pre **(A)** and post **(B)** headshake in an VM patient in the acute phase. There was an increase of 0.165 in the gain after the head oscillation (“headshaking”).

Another explanation for the lack of change in VOR gain following headshaking is that the VM patients included in the study were in an inter-ictal phase. Whilst both perceptual and cognitive deficits have been reported in the inter-ictal phase in VM, and also patients with episodic vertigo from inner ear pathologies, changes in VOR gain may be more pronounced in the acute, ictal, phase (25). Indeed, in a single patient that was tested acutely (Figure 3), we observed an increase in VOR gain POST-HS that was >2.5 standard deviations of the mean POST-HS VOR gain of chronic VM patients in our cohort.

In this study, we measured VOR gain using vHIT as a simple method of recording VOR gain and a surrogate measure of vestibular excitability. Other studies may explore canal-otolith interactions, as dizziness in VM patients is provoked typically when the superior semicircular canals and otolith organs are stimulated simultaneously (9). Accordingly, the use of cervical or ocular vestibular evoked myogenic potentials (VEMP) instead of vHIT could give information on saccule and utricle vestibular function (26).

It is possible that a significant change in VOR gain between PRE- and POST-HS was not seen because the vHIT is a supra-threshold stimulus at high frequencies (above 5 Hz), so increases in gain are less likely when the VOR is already functioning at its optimal level (23), thus representing a physiological ceiling effect on the VOR. The fact that all VM patients but roughly half of the BPPV patients and none of the HC in our study referred feeling sick when reading in the passenger seat of a car and a heightened motion susceptibility supports the idea that head motion could be a variable of interest in the diagnosis of VM patients. Perhaps the use of a low frequency test of VOR function may be a more suitable stimulus, but not without its own limitations. Whilst patients with VM report nausea or motion sickness symptoms when reading in the passenger seat of a car, the MSSQ was not significantly different for the VM group compared to disease and healthy controls. That this specific compliant is more than four times more likely to be a factor in VM relative to BPPV suggests that a question specifically addressing concurrent visual and motion stimuli in the context of motion sickness (i.e., reading in a moving vehicle) may be more sensitive than a motion susceptibility questionnaire for VM.

Given that this study was sufficiently powered to detect an effect on VOR gain thresholds (7), physiological changes to the high-frequency VOR gain may be less likely to occur where the VOR may be functioning at ceiling. Future studies may need to explore the use of lower-frequency VOR stimuli to overcome this potential limitation.

## Conclusion

Patients with VM do not have heightened VOR gain immediately following headshake in the inter-ictal phase. Other studies may wish to apply this protocol in patients with acute VM or use visual motion stimuli instead of headshaking to alter visuo-vestibular interactions. Understanding the pathophysiological mechanisms of VM and development of simple clinical biomarkers are urgently needed to ensure timely and accurate diagnosis of one of the most common episodic vestibular disorders.

## Data availability statement

The raw data supporting the conclusions of this article will be made available by the authors, without undue reservation.

## Ethics statement

The studies involving human participants were reviewed and approved by Northwest - Greater Manchester South Research Ethics Committee. The patients/participants provided their written informed consent to participate in this study.

## Author contributions

PC, NK, QA, and DK: conceptualization. PP, SC, and LG: data collection. PP and PC: analysis and first draft. PC, QA, and DK: manuscript preparation and revision. All authors contributed to the article and approved the submitted version.

## Conflict of interest

The authors declare that the research was conducted in the absence of any commercial or financial relationships that could be construed as a potential conflict of interest.

## Publisher's note

All claims expressed in this article are solely those of the authors and do not necessarily represent those of their affiliated organizations, or those of the publisher, the editors and the reviewers. Any product that may be evaluated in this article, or claim that may be made by its manufacturer, is not guaranteed or endorsed by the publisher.
